# A Paper on Protracted Valvular Disease of the Heart

**Published:** 1855-07

**Authors:** John W. Corson


*A Paper on Protracted Valvular Disease of the Heart.* Read before the Society of Statistical Medicine. By John W. Corson, M. D.

Dr. Corson here collates the principal facts of forty-one cases of heart disease with reference to prognosis. He gives the following table of average duration:

AVERAGE DURATION.

| | |
|---|---|
| Nine cases of Aortic Obstruction, - - - | $10\frac{1}{2}$ years. |
| Eight do. of Aortic Obstruction and Regurgitation, | $8\frac{1}{4}$ " |
| Nine do. of Mitral Regurgitation, - - - - | $8\frac{1}{2}$ " |
| Seven do. of Aortic and Mitral, - - - | 10 |
| Six do. of Miscellaneous, - - - - - - | 6 |
| Three do. of right and left simultaneously, - - | 4 |

Dr. C. argues from these facts that we may discriminate as to the probability of life in different heart affections.

